# Prevalence, Molecular Detection, and *In Vitro* Elimination of Latent Viruses and Viroids in Pome Fruit Germplasm of Kazakhstan

**DOI:** 10.3390/v18070767

**Published:** 2026-07-13

**Authors:** Aigul Madenova, Dinara Kaldybayeva, Timur Turdiyev, Raigul Abdikarimova, Yerkenaz Kaiypzhan, Balnur Kabylbekova, Sagi Soltanbekov, Zhankeldy Aitymbet, Muhammad Azhar Nadeem, Faheem Shehzad Baloch

**Affiliations:** 1 *In vitro* Genetic Resources Laboratory, Kazakh Fruit and Vegetable Research Institute, Almaty 050060, Kazakhstan; madenova.a@mail.ru (A.M.);; 2 Institute of Natural Sciences and Geography, Abai Kazakh National Pedagogical University, Dostyk av., 13, Almaty 050010, Kazakhstan; 3 Department of Biotechnology, Faculty of Science, Mersin University, Çiftlikköy Kampüsü, 33343 Mersin, Türkiye; 4 Department of Biotechnology, Samarkand State University, Samarkand 140104, Uzbekistan; 5 Department of Genetics, Institute of Biochemistry, Sh. Rashidov Samarkand State University, Samarkand 140104, Uzbekistan

**Keywords:** latent viruses, viroids, pome fruits, RT-qPCR, cryotherapy, virus-free planting material, phytosanitary monitoring

## Abstract

Latent viral and viroid infections represent a major phytosanitary threat to pome fruit production because infected propagation material facilitates the long-term spread of pathogens in orchards. This study investigated the prevalence of viral and viroid pathogens in apple and pear orchards of southern and southeastern Kazakhstan and evaluated the efficiency of integrated *in vitro* sanitation approaches. A total of 34 samples collected from commercial orchards were analyzed using RT-qPCR. Apple stem pitting virus (ASPV) was the most prevalent pathogen detected across all surveyed regions, followed by Apple chlorotic leaf spot virus (ACLSV). Apple stem grooving virus (ASGV), Apple hammerhead viroid (AHVd), Apple mosaic virus (ApMV), and Apple rubbery wood virus 1 (ARWV-1) were detected at lower frequencies. Notably, ARWV-1 and AHVd were detected exclusively in the Almaty Region, representing their first molecular detection in Kazakhstan. Mixed infections were common, and visual symptoms did not consistently correlate with molecular detection results, emphasizing the importance of RT-qPCR for reliable phytosanitary diagnostics. Integrated sanitation methods combining thermotherapy, chemotherapy, cryotherapy, and meristem culture effectively eliminated viral and viroid infections from infected apple and pear genotypes. Ribavirin at 15 mg L^−1^ provided the optimal balance between antiviral activity and explant viability. Complete elimination of ACLSV, AHVd, ASPV, ASGV, ApMV, and ARWV-1 was achieved after combined treatment, while regenerated plants maintained stable micropropagation capacity. The obtained results demonstrate the necessity of routine molecular monitoring and support the development of virus-free certification systems for pome fruit crops in Kazakhstan.

## 1. Introduction

Pome fruit crops occupy an important position in Kazakhstan’s horticultural sector. However, the extensive use of vegetative propagation and uncertified planting material contributes to the spread of latent viral and viroid infections in commercial orchards. These pathogens frequently remain asymptomatic, allowing their unnoticed dissemination through propagation material and resulting in long-term reductions in productivity, fruit quality, and orchard longevity [1,2]. In perennial fruit crops, latent infections become particularly problematic because infected mother plants continuously serve as reservoirs of pathogens during clonal propagation cycles [3].

Among the most economically important viral pathogens of apple and pear are Apple stem pitting virus (ASPV), Apple chlorotic leaf spot virus (ACLSV), Apple stem grooving virus (ASGV), Apple mosaic virus (ApMV), and several recently described viruses and viroids, including Apple hammerhead viroid (AHVd) and Apple rubbery wood viruses (ARWV-1 and ARWV-2) [4,5]. These pathogens are widely distributed in major fruit-producing regions worldwide and are commonly associated with mixed infections, which complicate disease diagnosis and may intensify physiological damage in host plants [6,7]. Several studies have demonstrated that visual symptoms alone are insufficient for reliable detection because many infected trees remain symptomless under field conditions [8].

Sanitation of vegetatively propagated planting material is a key component of phytosanitary management in perennial and clonally propagated fruit crops. Various *in vitro* approaches, including meristem culture, thermotherapy, chemotherapy, shoot tip culture, micrografting, and cryotherapy, have been successfully applied for the elimination of viruses and viroids from numerous horticultural species, including apple, grapevine, citrus, stone fruits, potato, and other clonally propagated crops [9,10,11]. In apple, integrated sanitation strategies combining thermotherapy, chemotherapy, and cryotherapy with meristem culture have proven effective for eliminating latent viruses and viroids while preserving the regeneration capacity of valuable germplasm [10,12,13]. Similar approaches have also been successfully implemented in grapevine for the eradication of multiple viruses and viroids using thermotherapy combined with meristem tip micrografting or antiviral chemotherapy [9,11]. The efficiency of these sanitation methods depends on multiple factors, including host genotype, pathogen species, meristem size, physiological status of the explants, and treatment conditions. Therefore, sanitation protocols require optimization for each crop–pathogen combination before their routine implementation in certification and virus-free propagation programs.

In recent years, molecular diagnostic methods, particularly reverse transcription quantitative PCR (RT-qPCR), have become essential tools for phytosanitary monitoring and certification of planting material due to their high sensitivity and specificity [14]. These methods are increasingly used in international certification systems for the production of virus-free germplasm and nursery material [15].

Despite the economic importance of fruit production in Kazakhstan and substantial advances in sanitation technologies worldwide, information regarding the prevalence of economically important viruses and viroids in commercial apple and pear orchards remains limited [16,17]. The few available RT-qPCR-based studies have confirmed the occurrence of latent viral infections in commercial apple orchards, emphasizing the growing phytosanitary importance of molecular diagnostics for pome fruit crops [15].

Therefore, the aims of the present study were: (i) to evaluate the prevalence of major viral and viroid pathogens in apple and pear orchards of southern and southeastern Kazakhstan using RT-qPCR diagnostics, and (ii) to assess the efficiency of combined *in vitro* sanitation methods for the production of virus-free planting material. The novelty of this study lies in the combined assessment of latent viral and viroid infections in apple and pear germplasm from Kazakhstan together with the evaluation of integrated *in vitro* sanitation strategies for infected plant material. While previous studies have investigated molecular detection of pome fruit viruses and viroids or sanitation of infected apple material separately [5,10,18], comparable data from Kazakhstan and Central Asia remain scarce. To our knowledge, this is one of the first studies linking targeted molecular diagnostics, regional phytosanitary monitoring, and practical sanitation approaches for pome fruit germplasm in Kazakhstan. By integrating RT-qPCR-based molecular diagnostics with optimized sanitation protocols, the present study provides baseline data for the development of virus-free certification systems, improved phytosanitary management, and future molecular surveillance of apple and pear propagation material in Kazakhstan.

## 2. Materials and Methods

### 2.1. Plant Material

A total of 34 apple and pear samples were collected from commercial orchards located in the Almaty, Turkestan, and Zhambyl regions of Kazakhstan. Sampling was performed according to GOST 12430-2019 standards [19] for phytosanitary inspection and laboratory examination of agricultural products.

In the Almaty Region (Talgar District, Talgar Branch of KazFPRI LLC; 43°17′27″ N 77°12′15″ E), 12 apple cultivars (‘Idared’, ‘Golden Crown’, ‘Grushovka Moskovskaya’, ‘Pilot’, ‘Golden Delicious’, ‘Egemen’, ‘Rahat’, ‘Tyulpan’, ‘Talgarskoye’, ‘Jonagold’, ‘Belle Fleur Almatinka’, and ‘Zarja Alatau’) and four pear cultivars (‘Harrow Delight’, ‘Anjou’, ‘Noyabrskaya’, and ‘Talgarskaya Krasavitsa’) were analyzed.

In the Turkestan Region, samples were collected from three locations: Koktal Farm, Tulkubas District (42°33′32.288″ N 70°24′2.687″ E), where nine apple cultivars were sampled (‘Idared’, ‘Aport’, ‘Golden Delicious’, ‘Rosemary’, ‘Melba’, ‘Nafis’, ‘Starkrimson’, ‘Tyulpan’, and ‘Kandil Sinap’); Kazygurt District (41°36′7.637″ N 69°22′2.022″ E), where four apple cultivars (‘Pink Lady’, ‘Aport’, ‘Idared’, and ‘Starkrimson’) were analyzed; and the Saryagash Branch of KazFPRI LLC, Zhemisti Village (41°32′2.545″ N 69°21′36.069″ E), where two apple cultivars (‘Borovinka Tashkent’ and ‘Konfetnoye’) were sampled.

In the Zhambyl Region, samples were collected from Merke Village, Merken District (42°48.584′ N 73°10.387′ E), including the apple cultivars ‘Guzel’, ‘Konfetnoye’, and ‘Red Delicious’. All analyzed material originated exclusively from commercial orchards.

### 2.2. Assessment of Visual Symptoms

**Figure 1:**
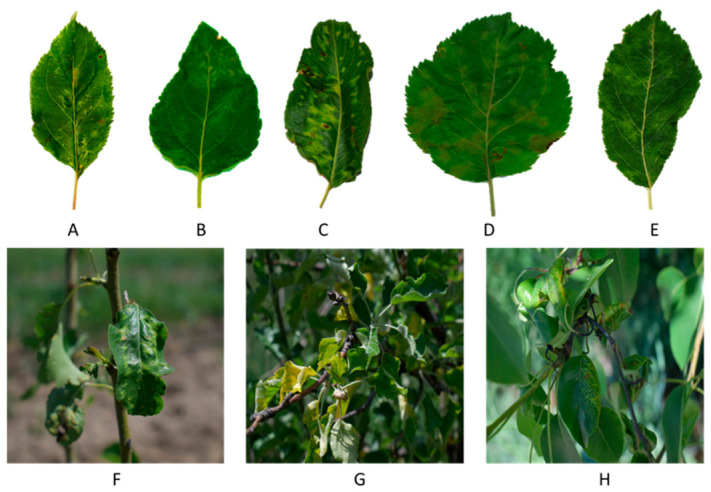
Visual symptoms of viral and viroid infections observed in apple and pear orchards of Kazakhstan: (**A**) mosaic symptoms; (**B**) vein chlorosis; (**C**) necrotic spots and leaf deformation; (**D**) marginal chlorosis and scorch; (**E**) marbling symptoms; (**F**) twisting of young shoots; (**G**) chlorosis and leaf dieback; (**H**) necrosis of leaves and shoot tips.

Leaves and young shoots were visually examined for symptoms associated with viral infections, including mosaic patterns, chlorosis, necrosis, leaf deformation, marginal scorch, and shoot twisting. Symptom severity and distribution were recorded prior to molecular analysis (Figure 1).

### 2.3. RNA Extraction and RT-qPCR Analysis

Total RNA was extracted from 200 mg of fresh or frozen leaf tissue using the Phyto-Sorb^®^ kit (SINTOL^®^, Moscow, Russia) according to the manufacturer’s instructions. Plant tissues were ground in liquid nitrogen and homogenized in extraction buffer. RNA concentration and purity were determined using a NanoDrop 2000 spectrophotometer (Thermo Fisher Scientific, Waltham, MA, USA). RNA samples were adjusted to a final concentration of 5 ng µL^−1^ and stored at −80 °C until analysis.

The diagnostic panel was designed to include the principal latent and economically important graft-transmissible viruses and viroids affecting apple and pear that are relevant to vegetative propagation, germplasm exchange, and certification systems. The selected targets comprised the widespread pome fruit viruses Apple chlorotic leaf spot virus (ACLSV), Apple stem pitting virus (ASPV), Apple stem grooving virus (ASGV), and Apple mosaic virus (ApMV), together with recently described or emerging pathogens, including Apple necrotic mosaic virus (ApNMV), Apple rubbery wood viruses 1 and 2 (ARWV-1 and ARWV-2), Apple green crinkle-associated virus (AGCaV), and Apple hammerhead viroid (AHVd). These pathogens were selected based on their documented phytosanitary importance, their inclusion in internationally recognized certification and diagnostic programs, particularly those recommended by EPPO, and their ability to remain latent, occur in mixed infections, and spread through infected vegetative propagation material [5,10,18].

Detection was performed using commercial multiplex RT-qPCR kits (LETGEN Biotechnology Laboratory Ltd., Izmir, Türkiye) containing pathogen-specific primers and TaqMan probes.

Each 20 µL RT-qPCR reaction mixture consisted of 10 µL qPCR Master Mix, 2 µL Primer–Probe Mix, 1 µL RTase Mix, 4 µL RNA template, and 3 µL nuclease-free water. Amplification was carried out on a Rotor-Gene Q thermocycler (QIAGEN, Hilden, Germany) under the following conditions: reverse transcription at 50 °C for 15 min, polymerase activation at 95 °C for 2 min, followed by 40 cycles of denaturation at 95 °C for 10 s and annealing/extension at 60 °C for 30 s.

### 2.4. In Vitro Sanitation Procedures

*In vitro* apple cultivars (‘Rahat’, ‘Zarja Alatau’, ‘El Rosa’, ‘Aport’, ‘Nafis’, ‘Jonagold’, and ‘Egemen’) and pear cultivars (‘Noyabrskaya’, ‘Anjou’, ‘Talgarskaya Krasavitsa’, and ‘Harrow Delight’) previously confirmed as infected by RT-qPCR were used for sanitation experiments.

Apical meristems were cultured on Murashige and Skoog (MS) medium supplemented with plant growth regulators. Apple cultures contained 0.8 mg L^−1^ BAP, 0.5 mg L^−1^ GA_3_, and 0.1 mg L^−1^ IAA, whereas pear cultures contained 1.0 mg L^−1^ BAP and 0.1 mg L^−1^ IAA.

Thermotherapy was performed at 37–38 °C for 20–30 days under a 16 h photoperiod. Meristematic tissues (0.2–0.5 mm) were subsequently excised and transferred to fresh culture medium. For chemotherapy, ribavirin was added at concentrations of 15 or 25 mg L^−1^. Cryotherapy was performed by vitrification of meristems followed by immersion in liquid nitrogen (−196 °C) and subsequent recovery under *in vitro* conditions.

Following sanitation treatments, regenerated plants were tested again by RT-qPCR to confirm elimination of viral and viroid pathogens. Genetic stability of regenerated material was evaluated using SSR markers CH03g07, CH04e03, CH05d11, and CH05e03.

### 2.5. Statistical Analysis

All experiments were performed in triplicate. Data are presented as mean ± standard deviation (SD). Statistical analysis was conducted using Duncan’s multiple range test at *p* ≤ 0.05 with IBM SPSS Statistics version 27.0 (IBM Corp., Armonk, NY, USA).

## 3. Results

### 3.1. Molecular Survey of Apple and Pear Orchards Using RT-qPCR

To assess the phytopathological status of apple orchards in the southern regions of Kazakhstan, a comparative study was conducted, including visual monitoring of plants for symptoms of viral diseases (Figure 1) and molecular diagnostics using real-time reverse transcription PCR (RT-qPCR) (Table 1). Molecular detection of viral pathogens was performed on apple and pear samples collected from five regions of Kazakhstan. Virus detection was accompanied by the evaluation of visual symptoms on leaves and shoots.

In the Almaty Region (Talgar District, Talgar Branch, KazFPRI orchards), visual symptoms of viral infection (spots, mosaic patterns, leaf deformation) were observed in 13 of 16 cultivars (81.25%). RT-qPCR analysis revealed that the most prevalent virus was ASPV (81.25%), followed by ACLSV (37.5%), ARWV-1 (25%), ApMV (25%), AHVd (18.75%), and ASGV (12.5%). The highest viral loads were detected in the cultivars ‘Egemen’, ‘Rahat’, and ‘Tyulpan’, which were simultaneously infected with multiple viral pathogens. Notably, ARWV-1 and AHVd were detected only in samples collected from the Almaty Region, representing the first molecular detection of these pathogens in Kazakhstan.

In the Tulkubas District (Koktal Farm, Turkestan Region), visual symptoms were observed in 4 of 9 cultivars (44.4%). RT-qPCR analysis showed high prevalence of ASPV (88.9%) and ACLSV (66.7%). ApMV was detected in a single sample (11.1%), whereas ASGV, ARWV-1, and AHVd were not detected in this region.

In the Kazygurt District (Turkestan Region), visual symptoms were observed in only one cultivar; however, all samples tested positive for ASPV (100%), indicating latent infection.

In the Saryagash District (Turkestan Region, Zhemisti Village, Saryagash Branch), visual symptoms were observed in one of the two cultivars, whereas RT-qPCR detected ASPV in all samples (100%).

In the Merken District (Zhambyl Region, Merke Village), no visual symptoms were observed; however, RT-qPCR revealed the presence of ASPV in all analyzed cultivars (100%).

**Figure 2:**
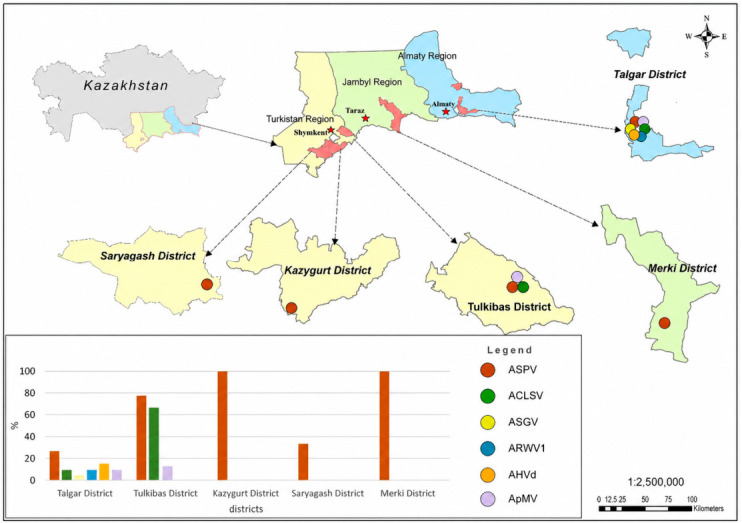
Map of virus and viroid surveys of pome fruit crops in southern and south-eastern Kazakhstan.

Thus, ASPV was the most prevalent virus across all surveyed regions, being detected in every district. ACLSV was also widely distributed, particularly in the Tulkubas District. Other pathogens (ASGV, AHVd, ApMV, ARWV-1) were mainly detected in the Almaty Region. Several asymptomatic cultivars tested positive in RT-qPCR analysis, demonstrating that visual assessment alone is insufficient for reliable phytosanitary evaluation of pome fruit orchards (Figure 2).

For the sanitation experiments, *in vitro* apple (*Malus domestica*) cultivars ‘Rahat’, ‘Zarja Alatau’, ‘El Rosa’, ‘Aport’, ‘Nafis’, ‘Jonagold’, ‘Egemen’ and pear (*Pyrus communis*) cultivars ‘Noyabrskaya’, ‘Anjou’, ‘Talgarskaya Krasavitsa’, and ‘Harrow Delight’, previously confirmed as infected with ApMV, ASGV, ARWV-1, ACLSV, ASPV, and AHVd by RT-qPCR (Table 1), were used.

### 3.2. Thermotherapy + Meristem Culture

Application of thermotherapy at 37–38 °C for 20–30 days, followed by cultivation of apical meristems, significantly improved virus elimination efficiency. Without prior thermotherapy, meristem culture efficiency ranged from 22.2% to 41.7%, with plant survival rates of 60–80%.

After combined thermotherapy and meristem culture, virus elimination rates increased as follows:

- ‘Noyabrskaya’—ApMV: 85.7%, ARWV-1: 71.4%;
- ‘Anjou’—ASGV: 75.0%;
- ‘Talgarskaya Krasavitsa’—ARWV-1: 71.4%;
- ‘Harrow Delight’—ARWV-1: 83.3%.

However, thermotherapy was associated with reduced plant survival (40–53.3%), reflecting genotypic sensitivity to heat stress (Table 2). These results confirm that virus elimination efficiency depends not only on the applied method but also on cultivar, type of viral infection, and the physiological condition of the explants.

### 3.3. Thermotherapy + Chemotherapy + Meristem Culture

To enhance the sanitation effect, ribavirin (15–25 mg L^−1^) was applied in combination with thermotherapy and meristem culture. After a 14-day acclimation period followed by thermotherapy at 36–38 °C, virus elimination efficiency reached 80–85% at a ribavirin concentration of 15 mg L^−1^, with explant survival rates of 65–80%.

Increasing the ribavirin concentration to 25 mg L^−1^ reduced survival to 50–70% and decreased the multiplication coefficient (Kr = 3.1–3.8), indicating phytotoxic effects of the compound. The cultivars ‘Egemen’ and ‘Noyabrskaya’ were most sensitive to stress, whereas ‘Jonagold’, ‘Anjou’, and ‘Talgarskaya Krasavitsa’ exhibited high survival rates (75–80%) and effective restoration of growth processes (Table 2).

Thus, a ribavirin concentration of 15 mg L^−1^ was determined to be optimal for *in vitro* sanitation, providing a balance between antiviral activity and plant viability.

### 3.4. Thermotherapy + Chemotherapy + Cryotherapy + Meristem Culture

To achieve complete elimination of viruses and viroids, a comprehensive method combining thermotherapy, chemotherapy, cryotherapy (meristem vitrification), and apical meristem culture was applied. Ultra-low temperatures (−196 °C) promoted the death of infected cells while preserving the viability of meristematic tissues.

**Figure 3:**
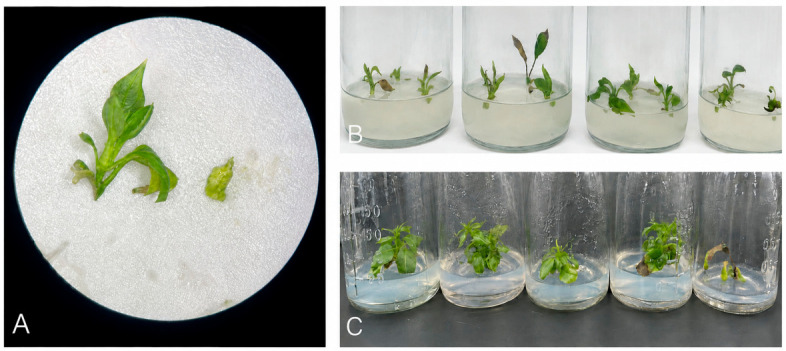
Sanitation of pome fruit crops using meristem culture: (**A**)—isolated meristem; (**B**)—regeneration of apple plants after *in vitro* thermotherapy; (**C**)—regeneration of pear plants after *in vitro* chemotherapy.

Following all treatment stages, PCR phytosanitary analysis confirmed the complete absence of viruses and viroids (ACLSV, AHVd, ASPV, ASGV, ApMV, ARWV-1) in all tested apple and pear genotypes (Table 2). Meristem viability after the multicomponent treatment decreased to 40–58%, while the multiplication coefficient (Kr) remained within 3.1–3.6, allowing subsequent micropropagation (Figure 3). The pear cultivar ‘Anjou’ exhibited the highest meristem survival (58%), whereas ‘Egemen’ and ‘Noyabrskaya’ were the least resilient (40–42%).

Post-sanitation control analysis confirmed the absence of detectable viruses in all regenerated apple and pear plants (Table 2). Additional SSR analysis verified the genetic uniformity of most cultivars. The exception was the apple cultivar ‘Aport’, which showed allele discrepancies at a single marker, potentially indicating chimerism (Table 2).

## 4. Discussion

Epidemiological significance of latent viral infections. The present study demonstrated the widespread occurrence of latent viral infections in commercial apple and pear orchards of southern and southeastern Kazakhstan, with ASPV representing the most prevalent pathogen across all surveyed regions. ACLSV was also frequently detected, whereas ASGV, AHVd, and ARWV-1 occurred less commonly and showed more localized distribution patterns. Similar epidemiological trends have been reported in Europe and Asia, where latent pome fruit viruses are widely disseminated through vegetative propagation systems and infected nursery material [6,7,8].

The high prevalence of ASPV observed in this study may be associated with the long-term use of uncertified planting material and the absence of a national virus-free certification system in Kazakhstan. Because apple and pear are clonally propagated perennial crops, latent viruses can accumulate and spread over multiple propagation cycles without visible symptom expression, contributing to the establishment of long-term infection reservoirs in orchards [3]. International exchange of rootstocks and scion material further increases the risk of introducing novel viral pathogens and genetic variants into local germplasm collections [4,17]. Recent studies on Kazakh apple germplasm have also demonstrated substantial genetic diversity and heterogeneous resistance profiles among local cultivars, emphasizing the importance of integrated phytosanitary and breeding strategies for sustainable orchard management [20,21].

The phytosanitary importance of latent infections extends beyond direct yield reduction. Chronic viral infections may alter photosynthetic performance, water-use efficiency, carbon allocation, and antioxidant metabolism, resulting in hidden productivity losses and reduced orchard longevity even in symptomless trees [22,23]. Therefore, the epidemiological situation revealed in Kazakhstan highlights the urgent need for systematic molecular monitoring and the establishment of virus-free propagation programs for pome fruit crops.

Latent infections and diagnostic implications. One of the most important findings of the present study was the inconsistency between visual symptom expression and RT-qPCR detection results. Several cultivars infected with ASPV, ACLSV, ASGV, AHVd, and ARWV-1 exhibited either weak symptoms or remained asymptomatic despite confirmed molecular detection. Similar discrepancies between symptom expression and infection status have also been reported in apple germplasm collections and commercial orchards in Europe and Asia, where molecular diagnostics revealed widespread latent infections in symptomless trees [24,25,26].

Latent infection behavior in woody hosts is influenced by multiple interacting biological factors. Viral distribution within perennial tissues is frequently uneven, leading to fluctuations in viral titer depending on leaf age, vascular transport, seasonal metabolic activity, and environmental conditions [23]. Furthermore, symptom expression is strongly modulated by temperature, drought stress, nutrient imbalance, and host physiological condition. Under favorable conditions, antiviral defense mechanisms such as RNA silencing may suppress viral replication sufficiently to maintain asymptomatic infections, whereas environmental stress may trigger rapid viral accumulation and visible disease symptoms [8,22].

Host genotype also plays a critical role in determining symptom severity and viral accumulation. The substantial genetic variability previously identified within Kazakhstan apple germplasm collections may partly explain the cultivar-dependent differences in symptom expression, stress tolerance, and regeneration capacity observed in the present study [27]. Certain cultivars tolerate systemic infections with minimal physiological damage, whereas susceptible genotypes exhibit chlorosis, mosaic symptoms, growth inhibition, or necrosis even at comparatively lower viral loads [28]. The cultivar-dependent variability observed in our study supports the hypothesis that genetic background strongly influences host–virus interactions in pome fruit crops.

These findings confirm that symptom-based diagnosis alone is insufficient for reliable phytosanitary evaluation of perennial orchards. Sensitive molecular approaches such as RT-qPCR are therefore essential for early detection, certification systems, and long-term phytosanitary management of propagation material [14,15].

Importance of mixed infections. Mixed infections were frequently observed in the analyzed orchards, particularly involving ASPV, ACLSV, ASGV, and AHVd. Such co-infections are increasingly recognized as important factors influencing disease epidemiology, viral pathogenicity, and host physiological responses in perennial fruit crops. Similar complex virome compositions and latent mixed infections have been reported in apple orchards and germplasm collections in several countries using molecular and high-throughput sequencing approaches [26,29,30].

Interactions among multiple viruses and viroids may result in synergistic effects that enhance viral replication, suppress host RNA silencing pathways, alter tissue tropism, and increase systemic movement within the host plant [14]. Consequently, mixed infections may intensify oxidative stress, disrupt chloroplast integrity, impair photosynthetic efficiency, and reduce plant productivity even in the absence of severe external symptoms [22]. Conversely, antagonistic interactions among pathogens may partially suppress symptom expression, further complicating field diagnostics.

In the present study, several cultivars carrying mixed infections exhibited inconsistent visual phenotypes despite positive RT-qPCR results, supporting the hypothesis that virus–virus and virus–host interactions substantially influence disease expression. Similar observations have been reported in apple orchards and germplasm collections from Europe and Asia, where molecular analyses revealed latent co-infections associated with long-term physiological decline and reduced orchard performance [30,31].

The biological complexity of mixed infections represents an important challenge for phytosanitary systems because infected propagation material may remain symptomless while simultaneously acting as a reservoir for multiple pathogens. Therefore, routine multiplex molecular diagnostics should become an integral component of certification and nursery management programs.

Comparative efficiency of sanitation approaches. The present study demonstrated that integrated *in vitro* sanitation approaches combining thermotherapy, chemotherapy, cryotherapy, and meristem culture were highly effective for eliminating latent viral pathogens from apple and pear germplasm. Among the evaluated methods, the multicomponent treatment strategy produced the highest sanitation efficiency, resulting in complete elimination of detectable viral RNA and viroid-associated signals in regenerated plants.

Thermotherapy likely reduced viral replication by disrupting viral RNA synthesis and systemic movement within infected tissues, thereby increasing the probability of recovering virus-free meristematic cells [10,32]. The antiviral effect of ribavirin may be associated with its role as a nucleoside analog interfering with RNA-dependent RNA polymerase activity and viral genome replication. However, increasing ribavirin concentration to 25 mg L^−1^ negatively affected explant survival and multiplication rate, indicating phytotoxic effects consistent with previous reports [33].

Cryotherapy further enhanced sanitation efficiency by selectively destroying vacuolated and highly differentiated infected cells while preserving the viability of small meristematic domains that are frequently virus-free due to limited vascular connectivity [10]. Nevertheless, sanitation efficiency remained genotype-dependent. Cultivars such as ‘Talgarskaya Krasavitsa’ and ‘Anjou’ demonstrated relatively high tolerance to combined stress treatments, whereas ‘Egemen’ and ‘Noyabrskaya’ exhibited lower survival rates. These differences may reflect cultivar-specific variation in regeneration potential, stress tolerance, meristem size, and viral load.

The observed relationship between mixed infections and reduced explant survival further suggests that pathogen complexity may influence sanitation success. Plants carrying multiple viruses may experience elevated oxidative stress and metabolic instability during thermotherapy and cryotherapy, reducing regeneration capacity and increasing tissue sensitivity.

Implications for Kazakhstan certification systems. The results of this study have important implications for the future development of national certification and phytosanitary systems for pome fruit crops in Kazakhstan. At present, the country lacks a comprehensive infrastructure for the production and monitoring of certified virus-free planting material, despite the extensive use of vegetative propagation systems in commercial orchards.

The widespread occurrence of latent infections identified in this study demonstrates that visual inspection alone cannot ensure phytosanitary safety of nursery material. The implementation of routine RT-qPCR screening protocols, combined with periodic high-throughput sequencing (HTS)-based surveillance, would significantly improve the reliability of pathogen detection and reduce the risk of distributing infected propagation material [34,35].

In addition, integrated sanitation protocols combining thermotherapy, chemotherapy, cryotherapy, and meristem culture could serve as the basis for establishing national virus-free germplasm collections and certification programs. Similar systems have already been successfully implemented in Europe and North America and have significantly reduced the epidemiological burden of latent fruit tree viruses [15]. In the present study, several cultivars carrying mixed infections exhibited inconsistent visual phenotypes despite positive RT-qPCR results, supporting the hypothesis that virus–virus and virus–host interactions substantially influence disease expression. Similar observations have been reported in apple orchards and germplasm collections from Europe and Asia, where molecular analyses revealed latent co-infections associated with long-term physiological decline and reduced orchard performance [29,30,31].

Given the increasing international exchange of planting material and climate-driven changes in pathogen distribution, the establishment of standardized phytosanitary programs is becoming strategically important for the sustainable development and competitiveness of Kazakhstan’s fruit-growing sector. In addition, recent screening programs targeting disease-resistance traits in Kazakhstan apple cultivars highlight the strategic importance of integrating molecular breeding with virus-free certification approaches for the long-term improvement and conservation of local germplasm resources [20,21].

Limitations of the study. Despite the important findings obtained in this study, several limitations should be acknowledged. First, the number of analyzed samples was relatively limited and represented selected commercial orchards located in southern and southeastern Kazakhstan. Therefore, the results should be interpreted as a molecular survey of the investigated regions rather than a comprehensive epidemiological assessment of virus and viroid distribution throughout the country. Broader surveys including additional geographic regions, cultivars, and larger sample sizes will be necessary to obtain a more complete understanding of the epidemiology of pome fruit viruses and viroids in Kazakhstan.

Second, pathogen detection was based on targeted RT-qPCR assays designed to detect the major economically important viruses and viroids included in international certification programs. Consequently, the absence of amplification for other pathogens should not be interpreted as evidence that the tested plants were free from all possible viral or viroid agents. Additional known, divergent, or currently undescribed viruses and viroids may be present in apple and pear germplasm but remain undetected when using targeted diagnostic assays. High-throughput sequencing (HTS) has proven to be a powerful complementary approach for comprehensive virome characterization and for detecting viruses and viroids that may escape targeted molecular diagnostics [5,18]. Future HTS-based studies, together with sequence-based confirmation of selected detections, would provide a more complete characterization of the virome of Kazakhstan pome fruit germplasm, facilitate the identification of divergent isolates and novel pathogens, and further strengthen national phytosanitary monitoring and certification programs.

Third, seasonal fluctuations in pathogen titers were not evaluated. Because virus accumulation in perennial fruit crops may vary according to plant phenology and environmental conditions, longitudinal sampling throughout the growing season would improve the interpretation of latent infections and increase diagnostic reliability.

Finally, phylogenetic characterization of the detected viral isolates was beyond the scope of the present study. Sequence-based analyses would clarify the genetic diversity, evolutionary relationships, and possible geographic origins of virus and viroid populations occurring in Kazakhstan and would provide independent confirmation of the detection of recently described pathogens, including ARWV-1.

## 5. Conclusions

Phytosanitary monitoring of apple and pear orchards in southern and southeastern Kazakhstan demonstrated the occurrence of latent viral and viroid infections in the surveyed regions. ASPV was the most frequently detected pathogen and was identified in all surveyed regions, whereas ACLSV showed particularly high prevalence in the Tulkubas District. ASGV, AHVd, ApMV, and ARWV-1 were detected less frequently. AHVd and ARWV-1 were identified only in the Almaty Region and represent the first RT-qPCR-based molecular evidence of their occurrence in Kazakhstan.

The study demonstrated discrepancies between visual symptom expression and molecular detection results, confirming that RT-qPCR is essential for reliable phytosanitary diagnosis of perennial fruit crops. Mixed infections were frequently detected and may contribute to long-term physiological decline and reduced productivity of infected orchards.

Integrated sanitation approaches combining thermotherapy, chemotherapy, cryotherapy, and meristem culture proved effective for producing pathogen-tested planting material. Following multicomponent treatment, none of the targeted viruses or viroids were detected by RT-qPCR in regenerated plants, while stable micropropagation capacity was maintained. Ribavirin at 15 mg L^−1^ provided the best balance between antiviral activity and explant viability under the conditions evaluated in this study.

Overall, the results highlight the importance of routine molecular monitoring and provide baseline data supporting the future development of virus-free certification systems and phytosanitary management programs for apple and pear propagation material in Kazakhstan.

## Figures and Tables

**Table 1:** Prevalence of viral and viroid pathogens detected by RT-qPCR in apple and pear orchards of Kazakhstan.

Region/Location	Number of Samples	Prevalence of Viral and Viroid Pathogens, (%)					
		ASPV	ACLSV	ASGV	AHVd	ApMV	ARWV-1
Almaty Region (Talgar District)	16	81.3	37.5	12.5	18.8	25.0	25.0
Turkestan Region (Tulkubas District)	9	88.9	66.7	0	0	11.1	0
Turkestan Region (Kazygurt District)	4	100	0	0	0	0	0
Turkestan Region (Saryagash District)	2	100	0	0	0	0	0
Zhambyl Region (Merke District)	3	100	0	0	0	0	0

ASPV—Apple stem pitting virus; ACLSV—Apple chlorotic leaf spot virus; ASGV—Apple stem grooving virus; AHVd—Apple hammerhead viroid; ApMV—Apple mosaic virus; ARWV-1—Apple rubbery wood virus 1.

**Table 2:** Efficiency of combined *in vitro* sanitation methods for elimination of viral and viroid pathogens from apple and pear cultivars.

Sanitation Method	Cultivar/Genotype	Virus	Initial Explants (No.)	Virus Elimination (%)	Viability (%)	Multiplication Coefficient (Kr *)
Meristem Culture	Noyabrskaya	ApMV	15	40.0	66.7	3.6 ± 0.3
		ARWV-1	15	50.0	66.7	3.6 ± 0.3
	Anjou	ASGV	15	27.3	73.3	4.3 ± 0.3
	Talgarskaya Krasavitsa	ARWV-1	15	41.7	80.0	4.1 ± 0.3
	Harrow Delight	ARWV-1	15	22.2	60.0	4.0 ± 0.3
Thermotherapy + Meristem Culture	Noyabrskaya	ApMV	15	85.7	46.7	3.6 ± 0.3
		ARWV-1	15	71.4	46.7	3.6 ± 0.3
	Anjou	ASGV	15	75.0	53.3	4.3 ± 0.3
	Talgarskaya Krasavitsa	ARWV-1	15	71.4	46.7	4.1 ± 0.3
	Harrow Delight	ARWV-1	15	83.3	40.0	4.0 ± 0.3
Chemotherapy + Thermotherapy + Meristem Culture (15 mg/L Ribavirin)	Jonagold	ACLSV	20	100	80	4.2 ± 0.3
	Egemen	ACLSV, AHVd, ASPV, ASGV, ApMV	20	100	65	3.8 ± 0.2
	Rahat	ACLSV, AHVd, ASPV, ASGV	20	100	70	3.9 ± 0.2
	Golden Delicious	ApMV	20	100	75	4.1 ± 0.3
	Aport	ACLSV, ASPV	20	100	70	3.7 ± 0.2
	Harrow Delight	ARWV-1	20	100	75	4.0 ± 0.3
	Noyabrskaya	ARWV-1, ApMV	20	100	70	3.6 ± 0.3
	Talgarskaya Krasavitsa	ARWV-1	20	100	75	4.1 ± 0.3
	Anjou	ASGV	20	100	80	4.3 ± 0.3
Chemotherapy + Thermotherapy + Cryotherapy + Meristem Culture	Jonagold	ACLSV	20	100	55	3.5 ± 0.2
	Egemen	ACLSV, AHVd, ASPV, ASGV, ApMV	20	100	40	3.1 ± 0.3
	Rahat	ACLSV, AHVd, ASPV, ASGV	20	100	45	3.2 ± 0.2
	Golden Delicious	ApMV	20	100	50	3.4 ± 0.3
	Aport	ACLSV, ASPV	20	100	48	3.3 ± 0.2
	Harrow Delight	ARWV-1	20	100	52	3.5 ± 0.2
	Noyabrskaya	ARWV-1, ApMV	20	100	42	3.1 ± 0.3
	Talgarskaya Krasavitsa	ARWV-1	20	100	50	3.4 ± 0.2
	Anjou	ASGV	20	100	58	3.6 ± 0.3

MC—meristem culture; thermotherapy was performed at 37–38 °C; chemotherapy included ribavirin treatment; cryotherapy involved vitrification and storage in liquid nitrogen (−196 °C). * Kr—multiplication coefficient of regenerated microshoots.

## Data Availability

The data presented in this study are available on request from the corresponding author.
